# CYP51A1 in health and disease: from sterol metabolism to regulated cell death

**DOI:** 10.1038/s41420-025-02621-7

**Published:** 2025-07-14

**Authors:** Fangquan Chen, RuiRui Liang, Jieting Zhang, Rui Kang, Daolin Tang, Jiao Liu

**Affiliations:** 1 DAMP Laboratory, The Third Affiliated Hospital, Guangzhou Medical University, Guangzhou, Guangdong China; 2 The Second Affiliated Hospital of Guangzhou Medical University, Guangzhou, Guangdong China; 3 Department of Surgery, UT Southwestern Medical Center, Dallas, TX USA

**Keywords:** Cell death, Cancer

## Abstract

How do cells precisely coordinate sterol metabolism with survival and death signals in diverse physiological and pathological contexts? This fundamental question has gained increasing attention as accumulating evidence reveals that enzymes traditionally associated with lipid biosynthesis may have unexpected regulatory functions beyond metabolism. Cytochrome P450 family 51 subfamily A member 1 (CYP51A1), a conserved sterol 14α-demethylase essential for cholesterol synthesis, exemplifies this emerging concept. Although well-characterized as an antifungal drug target in microorganisms, the roles of human CYP51A1 in development, cell death regulation, and disease pathogenesis remain underexplored. Recent studies have uncovered that CYP51A1 not only contributes to cholesterol homeostasis but also modulates multiple forms of regulated cell death—including apoptosis, ferroptosis, alkaliptosis, and pyroptosis—via sterol intermediates or cholesterol-independent mechanisms. Moreover, dysregulation of CYP51A1 has been implicated in a wide spectrum of diseases, such as cancer, cataracts, Antley-Bixler syndrome, autoimmune disorders, metabolic liver disease and neurodegeneration. In this review, we provide a comprehensive synthesis of CYP51A1’s structure, regulatory networks, and non-canonical functions. We propose a unifying framework in which CYP51A1 integrates metabolic reprogramming and cell fate control, highlighting its potential as a therapeutic target across diverse human diseases.

## Facts

1. CYP51A1 is a conserved sterol 14α-demethylase required for cholesterol synthesis. Its abnormal expression is associated with many human metabolic diseases (such as cancer, cataracts, Antley-Bixler syndrome, autoimmune disorders, metabolic liver disease and neurodegeneration)
2. The different regulatory cell death mechanisms of CYP51A1-mediated cancer cells are still unclear, and why CYP51A1-triggered cell death is different in different contexts is unclear.
3. In the future, in-depth exploration of the structure of CYP51A1, development of specific regulators, and elucidation of the cell death pathways regulated by CYP51A1 in different contexts will contribute to the treatment of various metabolic diseases and cancers.

## Introduction

Sterol metabolism is a fundamental biological process that supports the structural integrity of membranes and the production of essential bioactive molecules such as hormones, bile acids, and vitamins [1]. At the center of this process lies a highly conserved enzyme: cytochrome P450 family 51 subfamily A member 1 (CYP51A1). As the only cytochrome P450 enzyme involved in all known sterol biosynthetic pathways across eukaryotes, CYP51A1 performs a critical demethylation step that enables the formation of cholesterol in mammals and ergosterol in fungi. This evolutionary conservation underscores its indispensable role in cellular physiology [2].

For decades, CYP51A1 has been studied primarily in the context of antifungal therapy, where its inhibition disrupts fungal membrane synthesis. However, emerging evidence suggests that in mammals—including humans—CYP51A1 serves functions far beyond sterol metabolism. Recent studies reveal its involvement in developmental processes (e.g., spermatogenesis and neural differentiation) [3, 4], immune regulation [5], and various forms of regulated cell death, such as apoptosis and ferroptosis [6, 7]. Furthermore, aberrant expression or function of CYP51A1 has been linked to multiple human diseases, including cancer, congenital syndromes, autoimmune conditions, metabolic liver disease, and neurodegenerative disorders.

Given its intersection with metabolism, cell fate determination, and disease pathogenesis, CYP51A1 represents a compelling yet underappreciated node in human biology. In this review, we aim to (1) provide a comprehensive overview of the structure and regulation of CYP51A1, (2) highlight its diverse roles in cellular homeostasis and death pathways, and (3) discuss its translational potential as a therapeutic target. By integrating insights from biochemistry, cell biology, immunology, and medicine, we hope to position CYP51A1 as a focal point for future cross-disciplinary research.

## Discovery of CYP51A1

Cytochrome P450 (CYP450) enzymes constitute a superfamily of heme-containing monooxygenases that mediate the synthesis and metabolism of lipids, hormones, and xenobiotics. The human genome encodes 57 CYP genes, including several non-coding pseudogenes, which are classified into 18 subfamilies (Table 1) [8]. Among them, the CYP2 subfamily is the largest, comprising 36 members, of which only 16 encode catalytically active enzymes. Human CYP enzymes are generally categorized based on their subcellular localization and function, falling into two major groups: microsomal and mitochondrial CYPs [9]. Of these, eight CYP enzymes participate in cholesterol metabolism (CYP7A1, CYP27A1, CYP46A1, CYP11A1, CYP51A1, CYP39A1, CYP8B1 and CYP7B1), but CYP51A1 is the only one involved in the de novo synthesis of cholesterol.

**Table 1:** Functional roles of human cytochrome P450 enzymes across different families.

CYP family	Family members	Functions	Refs.
1	Two subfamilies: A and B: CYP1A1, CYP1A2, CYP1B1	CYP1A1 is a major extrahepatic cytochrome P450 enzyme that converts a variety of substances to carcinogenic derivatives; CYP1A2 has monooxygenase and cyclooxygenase activities; and CYP1B1 metabolizes polycyclic aromatic hydrocarbons to carcinogenic compounds	[103, 104]
2	Eleven subfamilies: A, B, C, D, E, F, J, R, S, U, W: CYP2A6, CYP2A7, CYP2A13, CYP2B6, CYP2C8, CYP2C9, CYP2C18, CYP2C19, CYP2D6, CYP2E1, CYP2F1, CYP2J2, CYP2R1, CYP2S1, CYP2U1, CYP2W1	Total number of genes in the CYP2 family is 36 with 16 expressing active enzymes and the rest encoding pseudogenes. CYP2A6 and CYP2B6 are involved in the metabolism of nicotine and some drugs; CYP2C8 has the function of oxygenase; CYP2C9 is the main drug and xenobiotic metabolic enzyme in the liver; CYP2C19 can metabolize a variety of therapeutic drugs; CYP2D6 is responsible for the metabolism of 20–25% of commonly used prescription drugs; CYP2J2 is a major enzyme responsible for the conversion of polyunsaturated fatty acids into bioactive signaling lipids; CYP2R1 converts vitamin D3 in the liver to 25-hydroxyvitamin D3, which is then released into the bloodstream; CYP2U1 is a hydroxylase; CYP2R1 may be involved in arachidonic acid metabolism and vitamin D activation; CYP2E1 metabolizes exogenous toxins such as ethanol; the function of other CYP2s enzymes is not clear, CYP2 family and CYP3A4 (metabolize more than 50% of drugs) synergistic effect.	[105]
3	CYP3A4, CYP3A5, CYP3A7, CYP3A43	CYP3A4 is a glucocorticoid-induced enzyme; CYP3A5 and CYP3A4 are responsible for 50% of drug metabolism mediated by cytochrome P450 enzymes. CYP3A7 can hydroxylate testosterone and 3-dehydroepiandrosterone sulfate; CYP3A43 showed a low level of testosterone 6β-hydroxylase activity	[106–109]
4	Six subfamilies: A, B, F, V, X, Z: CYP4A11, CYP4A22, CYP4B1, CYP4F2, CYP4F3, CYP4F8, CYP4F11, CYP4F12, CYP4F22, CYP4V2, CYP4X1, CYP4Z1	There are 38 genes in the CYP4 family, of which 11 express active enzymes and the rest encode pseudogenes. CYP4A11, CYP4F2, CYP4F3, CYP4F8, CYP4F11, CYP4F12 and CYP4F22 ω-hydroxylate various lipid molecules; CYP4V2, CYP4X1, CYP4Z1 and other members have shown catalytic activity in some studies, but the specific functions still need to be further confirmed.	[110]
5	CYP5A1	CYP5A1 catalyzes the conversion of prostaglandin H2 to thromboxane A2 (TXA2)	[111]
7	CYP7A1, CYP7B1	Involved in bile acid synthesis	[112]
8	CYP8A1, CYP8B1	CYP8A1 catalyzes the conversion of prostaglandin H2 to prostacyclin; CYP8B1 is involved in bile acid synthesis	[113, 114]
11	Two subfamilies: A and B: CYP11A1, CYP11B1, CYP11B2	CYP11A1, CYP11B1 and CYP11B2 are mitochondrial enzymes that catalyze the synthesis and transformation of steroid hormones	[115]
17	CYP17A1	It has two different activities and participates in the synthesis of steroid hormones	[116]
19	CYP19A1	Involved in steroid hormone synthesis	[117]
20	CYP20A1	May have neurophysiologic effects	[118]
21	CYP21A2	Converts progesterone to 11-deoxycorticosterone and the conversion of 17-hydroxyprogesterone to 11-deoxycortisol	[117]
24	CYP24A1	A mitochondrial enzyme responsible for the degradation of 1,25-dihydroxyvitamin D3	[119]
26	Three subfamilies: A, B, and C: CYP26A1, CYP26B1, CYP26C1	Regulation of retinoic acid homeostasis	[120]
27	Three subfamilies: A, B, and C: CYP27A1, CYP27B1, CYP27C1	CYP27A1 is involved in the conversion of cholesterol into bile acids through a secondary (acidic) pathway; CYP27B1 converts 25-hydroxyvitamin D3 to 1,25-dihydroxyvitamin D3	[121]
39	CYP39A1	Preferred substrate is 24-hydroxycholesterol which is a major product of CYP46A1	[122]
46	CYP46A1	Expressed primarily in neurons of the central nervous system where it plays an important role in metabolism of cholesterol in the brain; 24S-hydroxycholesterol is a potent activator of LXR	[123]
51	CYP51A1	Catalyzes the removal of the 14α-methyl group from lanosterol in the cholesterol biosynthesis pathway	[68]

The discovery of CYP51A1 dates back to the mid-1970s, when studies using radiolabeled lanosterol in yeast and rat liver microsomes demonstrated the release of [14 C] formic acid, enabling a straightforward assay of lanosterol 14α-demethylase activity across species [10]. In 1991, Judy L. Raucy identified this demethylase activity in human liver, kidney, and lymphocytes, confirming its inhibition by classical antifungal agents such as ketoconazole and miconazole, and implicating its role in cellular cholesterol synthesis [11]. By 1993, Yoshiko Sonoda successfully purified the human CYP51A1 protein from liver microsomes and demonstrated its catalytic activity upon reconstitution with NADPH cytochrome P450 reductase (3.20 mmol per mg of protein /min), identifying its molecular weight as approximately 53 kDa [12]. This was followed by Yuri Aoyama’s 1996 landmark work, which characterized the full-length amino acid sequences of human and rat sterol 14-demethylase and revealed a high degree of homology with the fungal CYP51 enzyme. The 93% amino acid identity between human and rat orthologs suggested that CYP51A1 functions as a highly conserved housekeeping enzyme essential for survival [13, 14].

Concurrently, Damjana Rozman mapped the human *CYP51A1* gene and elucidated its genomic structure [15, 16]. Expression analyses by M. Strömstedt further demonstrated that CYP51A1 is widely expressed in human tissues, with highest levels in steroidogenic organs such as the testis, ovary, adrenal gland, liver, and prostate. In cell-based studies, CYP51A1 expression in human adrenocortical H295R cells was found to be downregulated by 25-hydroxycholesterol, a feedback inhibitor of cholesterol synthesis. Interestingly, this regulation was not observed in hepatocellular carcinoma HepG2 cells, suggesting cell-type-specific mechanisms and potential vulnerability of cancer cells to sterol metabolite modulation [17].

Further research by Strömstedt in 1998 revealed that post-meiotic male germ cells express CYP51A1 and synthesize meiosis-activating sterols at levels possibly exceeding those required for cholesterol production. Whether these sterols act as developmental signals or serve other unknown roles in spermatogenesis remains an open question [18].

Subsequent clinical studies have shown elevated CYP51A1 expression in primary ovarian and colorectal cancers, correlating with poorer prognosis [19, 20]. In parallel, small-molecule inhibitors targeting human CYP51A1 have been developed and tested in hepatocellular carcinoma models, supporting its therapeutic relevance [21].

In summary, the discovery of CYP51A1 as a conserved sterol 14α-demethylase involved in cholesterol biosynthesis was achieved through decades of biochemical, genetic, and structural studies across species.

## Structure of CYP51A1

**Fig. 1:**
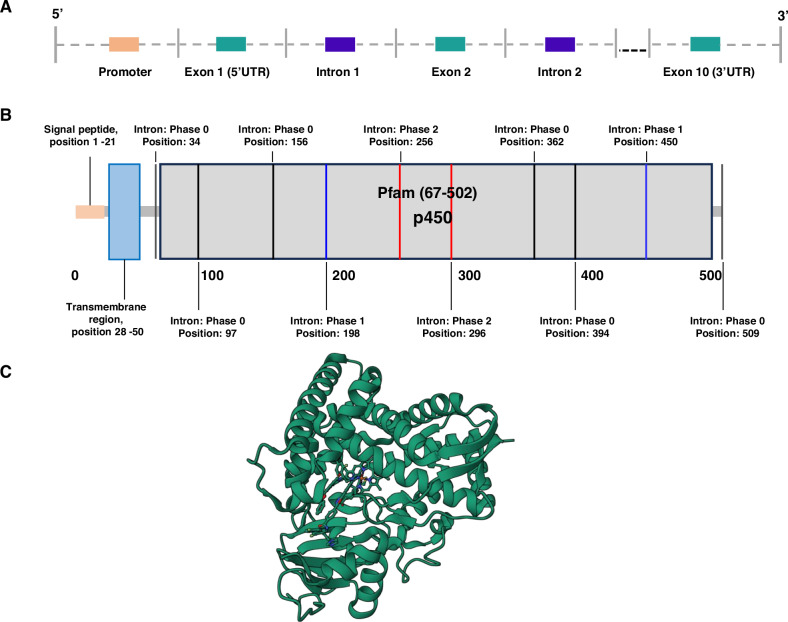
Gene structure, protein domains, and 3D structure of human CYP51A1. **A** Schematic representation of the CYP51A1 gene structure, showing the promoter region, exons (green), and introns (purple). The gene consists of 10 exons, with Exon 1 and Exon 10 contributing to the 5′ and 3′ untranslated regions (UTRs), respectively. **B** Domain architecture of the CYP51A1 protein, highlighting the signal peptide (residues 1–21) and the transmembrane region (residues 28–50) at the N-terminus. The conserved cytochrome P450 domain (Pfam 67–502) is shaded in gray. Positions of intron–exon boundaries and their associated splicing phases are indicated along the linear protein structure. **C** Ribbon diagram of the CYP51A1 3D structure (PDB ID: 4UHL), showing the binding of the ligand to the α-helix structure. This structure provides insights into the active site and potential ligand-binding regions critical for its sterol 14α-demethylase activity.

The structural characterization of CYP51A1 has provided critical insights into its enzymatic function and evolutionary conservation. In 1996, Rozman identified the functional human *CYP51A1* gene on chromosome 7q21.2-21.3 using DNA isolated from 19 somatic hybridization groups and a series of chromosome 7-specific yeast artificial chromosomes (YACs) [15, 16]. The gene spans 22 kb and is divided into 10 exons with an open reading frame of 1527 bp (Fig. 1A). TATA and CAAT are absent in human *CYP51A1*, but the 5-prime of intron 1 is enriched with ~64.7% GC and is contained in a CpG island [22]. CpG cytosines in these regions are not methylated and are more readily available to transcription factors such as the GC cassette, the cAMP-responsive element (CRE) [23], the sterol regulatory element (SRE), the GCAAT sequence (potential nuclear factor Y binding site), promoter elements and downstream promoter elements (DPE). The 3-prime UTR includes an Alu repeat and three possible polyadenylation sites [16]. Primer extension studies show that in liver, kidney, lung and placenta, the major transcription start sites are located 250 and 249 bp upstream of the translation start site and 100 bp upstream of the second major site, encoding a protein with 509 amino acids [16]. In addition to elements known to have regulatory roles, the promoter of the mammalian *CYP51A1* gene, including human, has three highly conserved regions of unknown function (downstream TFIID-binding element 1 [DBE1], DBE2, and DBE3), and these results suggest that the human *CYP51A1* is a housekeeping gene [17].

The existence of a soluble form of CYP51A1 was confirmed through the genomic structure of *Mycobacterium tuberculosis* (MT) in 1998 [24]. Subsequent studies have shown that CYP51A1 has two unique features compared to other P450 structures (Fig. 1B, C). The first is that the longest I-helix in the CYP450 molecule is broken up into two parts, the N-terminal part pulling away from the center of the molecule, and the other is the position of the B-C loop at the top of the molecule producing an open substrate access channel [25]. In eukaryotes, CYP51A1 binds to the lipid bilayer at its N-terminus and is associated with the endoplasmic reticulum (ER). The intact CYP51A1 protein contains an ordered amphipathic helix at the N-terminus in addition to a transmembrane helix, which directs the orientation of the catalytic unit in the lipid bilayer. The human CYP51A1 protein contains 509 amino acid residues and has a molecular weight of approximately 57278 Da. It contains a heme group, which is essential for the catalytic activity of the cytochrome P450 enzyme. The structure of the CYP51A1 protein contains multiple helices and β-folds, forming a complex three-dimensional structure, which facilitates the binding of the protein to the substrate and the catalysis of the reaction [26, 27]. As a cytochrome P450 enzyme, CYP51A1 protein catalyzes the oxidation of substrates in the cell, converting the substrate into biologically active products and maintaining the normal metabolism of the cholesterol synthesis pathway [28].

Collectively, CYP51A1 exhibits a specialized structural configuration that reflects its evolutionary adaptation to sterol metabolism. Understanding these structural elements not only illuminates its catalytic mechanism but also provides a basis for rational drug design targeting cholesterol-related diseases.

## Modulation of CYP51A1

**Fig. 2:**
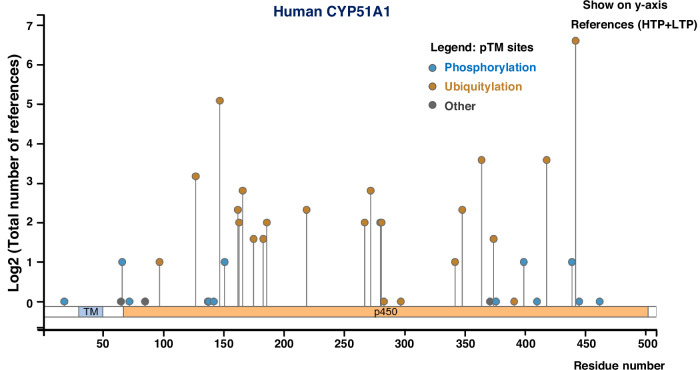
Post-translational modification landscape of human CYP51A1. This figure illustrates the distribution and frequency of experimentally validated post-translational modifications (PTMs) along the amino acid sequence of human CYP51A1. The x-axis denotes residue position, and the y-axis indicates the log2 (total number of references) reporting a specific P 509 amino acids TM site (from both high-throughput and low-throughput studies). PTMs are color-coded by type: phosphorylation (blue), ubiquitylation (orange), and other modifications (gray). Functional domains are annotated at the bottom: the transmembrane region (TM) and the conserved cytochrome P450 catalytic domain (p450). Notably, several high-frequency ubiquitylation sites are located within the p450 domain, suggesting possible regulation of CYP51A1 activity or stability via the ubiquitin-proteasome system (Data from https://www.phosphosite.org/).

The expression and activity of CYP51A1 are tightly regulated through both transcriptional mechanisms and post-translational modifications (PTMs), including ubiquitin-mediated degradation and protein–protein interactions (PPI) (Fig. 2). These regulatory pathways collectively govern CYP51A1 abundance, enzymatic function, and stability, thereby influencing its roles in physiological processes and disease pathogenesis.

### Transcriptional machinery

Mammalian *CYP51A1* mRNA expression is regulated by several factors, including transcription factors and microRNAs (miRNAs). The promoter region of the *CYP51A1* gene contains several transcription factors binding sites, such as sterol regulatory element binding transcription factors (SREBFs) [29], nuclear receptor subfamily 1 group H (NR1H; also known as LXR), peroxisome proliferator-activated receptor (PPAR), and one cut homeobox 1 (ONECUT1; also known as HNF6) [30, 31], all of which contribute to the modulation of gene expression under physiological and pathological conditions.

For example, when intracellular cholesterol levels are lowered, SREBF2 is activated and translocates to the nucleus where it binds to the sterol regulatory element (SRE) within the CYP51A1 promoter to enhance its transcription. This regulatory mechanism contributes to diverse biological processes—including lipid synthesis, immune modulation, and cell proliferation—and plays a critical role in the pathogenesis of coronary artery disease, autoimmune disorders, Antley-Bixler syndrome, and cancer [32–35]. In contrast, cholesterol derivatives negatively regulate CYP51A1 expression. For instance, 25-hydroxycholesterol suppresses *CYP51A1* mRNA levels in both human adrenocortical H295R and hepatocellular carcinoma HepG2 cells under cholesterol-deprived conditions [17]. Similarly, dietary supplementation with 1% cholesterol reduces hepatic *CYP51A1* mRNA and protein levels in Sprague-Dawley rats. This downregulation leads to the accumulation of the intermediate 3β-hydroxy-lanost-8-en-32-al, a known translational repressor of 3-hydroxy-3-methylglutaryl-CoA reductase (HMGCR). Notably, dietary addition of the HMGCR inhibitor lovastatin (0.02%) restores CYP51A1 expression, suggesting that CYP51A1 is an important component of the feedback loop regulating hepatic cholesterol biosynthesis via HMGCR modulation [36].

The human and mouse *CYP51A1* promoters contain a CRE element in addition to the SRE element, suggesting two major regulatory pathways-sterol-dependent regulation and cAMP-dependent regulation. Sterol feedback regulation is characteristic of all genes involved in cholesterol biosynthesis and homeostasis, whereas cAMP-dependent regulation is unique, suggesting that CYP51A1 may play a tissue-specific role distinct from cholesterol biosynthesis. For example, in the mouse testis, CYP51A1 expression is primarily regulated by the cAMP-responsive element modulator tau (CREMtau), which drives a germ cell-specific increase in *CYP51A1* mRNA levels during spermatogenesis. The *Crem* knockout (*Crem*^-/-^) mice, this upregulation is abolished in germ cells, while somatic expression of CYP51A1 remains unaffected. Interestingly, this regulatory mechanism is selective, as other cholesterol biosynthesis genes, such as squalene synthase, are not regulated by CREMtau during spermatogenesis [23]. Supporting this, promoter analysis of the human *CYP51A1* gene in JEG-3 cells—engineered to overexpress sterol regulatory element-binding protein 1a (SREBF1a)—confirmed the presence of multiple regulatory motifs, including a GC box, a cAMP response element (CRE), and a sterol regulatory element (SRE), indicating that human *CYP51A1* transcription is co-regulated by both cAMP- and sterol-dependent pathways [37].

In contrast, several regulatory factors can inhibit CYP51A1 expression through different pathways. For example, overexpression of the transcription factor forkhead box o4 (FOXO4) in 3T3L1 cells inhibits *CYP51A1* mRNA levels, leading to the intracellular accumulation of 24,25-dihydrolanosterol (DHL) and lanosterol. Notably, DHL—but not lanosterol—potently activates liver X receptor alpha (LXRα), suggesting that FOXO4 modulates cholesterol metabolism by enhancing DHL-mediated LXRα signaling. However, it remains unclear whether FOXO4 selectively regulates CYP51A1, or if its effects extend to other genes downstream in the cholesterol biosynthesis pathway [38].

In addition, cholesterol biosynthesis is subject to negative feedback by its end products. For instance, LXRα, a nuclear receptor for oxysterols, represses the expression of CYP51A1 and farnesyl-diphosphate farnesyltransferase 1 (FDFT1) in human HepG2 and HEK293 cells by binding to negative LXR response elements (nLXREs) in their promoters [39]. Post-transcriptional regulation also contributes to CYP51A1 repression. MicroRNA-219 (miR-219) downregulates *Cyp51a1* and *Srebf1* expression in CG-4 glial precursor cells derived from virus-infected mice and rats, resulting in suppressed cholesterol biosynthesis and improvement in clinical symptoms of demyelinating disease. Although these effects were observed in the context of *Theiler’s murine encephalomyelitis virus* (TMEV) BeAn strain infection, it is possible that similar regulatory mechanisms may apply in response to other viral infections [40].

Together, these findings demonstrate that CYP51A1 transcription is controlled by a complex interplay of sterol-dependent, cAMP-responsive, and inhibitory pathways, raising important questions about how these signals are integrated in tissue-specific and disease contexts.

### Post-translational modifications

Ubiquitination regulates protein stability, localization, and function by covalently attaching ubiquitin to lysine residues on target proteins. This process occurs through a highly coordinated enzymatic cascade involving three steps: (1) activation of ubiquitin by the E1 ubiquitin-activating enzyme, (2) transfer of ubiquitin to the E2 ubiquitin-conjugating enzyme, and (3) substrate-specific ligation of ubiquitin by an E3 ubiquitin ligase [41]. In response to different external stresses, aberrant ubiquitination of CYP51A1 may contribute to stress adaptation or disease pathogenesis.

For example, a study employing tandem ubiquitin-binding entity (TUBE) proteomics analyzed ubiquitination profiles in HEK293T cells exposed to five types of cellular stress—heat shock, oxidative stress, osmotic stress, UV irradiation, and proteasome inhibition. Among the proteins identified, epiplakin 1 (EPPK1), GCN1 (activator of EIF2AK4), and CYP51A1 were found to be stabilized under heat shock conditions upon bortezomib treatment, indicating their targeting for proteasomal degradation under stress. This stress-induced ubiquitination did not reflect nonspecific degradation of damaged proteins but rather a regulatory process involving stress granule disassembly and reactivation of cellular homeostasis after stress removal [42].

Additionally, nitric oxide (NO) induces rapid post-translational downregulation of CYP51A1 protein in human Huh7 hepatocellular carcinoma cells. This degradation is partially inhibited by bortezomib, suggesting a role for the ubiquitin-proteasome system, although only low levels of ubiquitination are detected. These findings imply a possible synergy between proteasomal and calpain-mediated degradation pathways. NO also modestly reduces *CYP51A1* mRNA expression, though the effect is less pronounced than the reduction at the protein level, further supporting a predominant role for post-translational control [43].

In addition to ubiquitination, CYP51A1 may also be regulated by phosphorylation, which could alter its enzymatic activity in steroid biosynthesis. While several phosphorylation sites have been identified, the functional consequences of these modifications remain poorly understood [44, 45].

In summary, post-translational modifications, particularly ubiquitination, play a critical role in controlling CYP51A1 expression and function under stress conditions. However, it remains unclear whether other modifications—such as sumoylation, mono-methylation, or phosphorylation—contribute to CYP51A1 regulation. Further investigation into these pathways may uncover novel mechanisms by which CYP51A1 contributes to disease development and cellular stress responses (Fig. 2).

### Protein-protein interaction

Aberrant expression or disruption of PPIs is a well-established driver of cellular dysfunction, contributing to the activation of cell death pathways and the development of various diseases [46]. CYP51A1 exerts its function through interactions with a network of regulatory and metabolic proteins.

A systems biology approach utilizing publicly available PPI databases and iSyTE lens gene expression data identified CYP51A1 as being directly connected to 51 proteins, of which 35 are expressed in the lens and 22 show lens-enriched expression. Notably, CYP51A1 interacts with cataract-associated proteins such as aldehyde dehydrogenase 1 family member A1 (ALDH1A1) and MAF BZIP transcription factor G (MAFG), highlighting its potential relevance to lens development and disease [47]. Additionally, CYP51A1 interacts with 15 core enzymes in the cholesterol biosynthesis pathway, including HMG-CoA reductase (HMGCR), FDFT1, dehydrocholesterol reductases (DHCRs), sigma non-opioid intracellular receptor 1 (SIGMAR1), and squalene epoxidase (SQLE), further supporting its central role in maintaining lipid homeostasis [47].

Functional studies in zebrafish have provided in vivo evidence that disruption of Pgrmc1 (progesterone receptor membrane component 1)—a membrane-associated protein that physically interacts with CYP51A1—leads to cataract formation, likely due to impaired CYP51A1 activity and dysregulated cholesterol metabolism [48]. In human cells, PGRMC1 deletion reduces CYP51A1 enzymatic activity, thereby suppressing cholesterol biosynthesis and promoting the accumulation of toxic sterol intermediates. Notably, PGRMC1 also binds other cytochrome P450 enzymes, such as cytochrome P450 family 3 subfamily A member 4 (CYP3A4), suggesting that individual variability in PGRMC1 function may influence broader drug metabolism pathways [49]. Emerging studies have further shown that CYP51A1 interacts with enzymes involved in lipid metabolism and participates in the regulation of cell death pathways, including ferroptosis and responses to SARS-CoV-2 infection, underscoring its involvement in both metabolic and immunopathological processes [50, 51].

In conclusion, PPIs substantially expand the functional repertoire of CYP51A1 beyond its canonical enzymatic role. However, whether these interactions contribute to biological functions independent of cholesterol biosynthesis remains unclear. Future studies aimed at mapping the interaction interfaces and identifying context-specific partners of CYP51A1 may offer new opportunities for therapeutic intervention.

## CYP51A1 in cell death

**Fig. 3:**
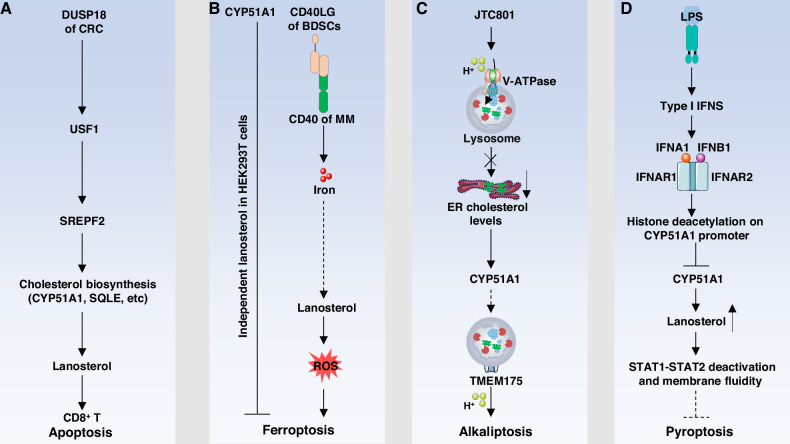
CYP51A1-mediated regulation of diverse regulated cell death pathways. **A** In CD8⁺ T cell apoptosis, DUSP18 of CRC activates the USF1–SREBF2 axis, inducing cholesterol biosynthetic enzymes including CYP51A1. Elevated lanosterol suppresses T cell activation and promotes apoptosis. **B** In ferroptosis, CD40 ligand (CD40LG) from bone marrow stromal cells activates CD40 on multiple myeloma (MM) cells, increasing intracellular iron and lanosterol, and lanosterol promotes ROS accumulation, enhancing ferroptotic cell death. Furthermore, the ferroptosis inhibitory function of CYP51A1 is independent of lanosterol in HEK293T cells. **C** In alkaliptosis, the small molecule JTC801 impairs lysosomal acidification by inhibiting V-ATPase, resulting in cholesterol accumulation in the ER and upregulation of CYP51A1. This process facilitates the degradation of lanosterol, restores TMEM175-mediated proton efflux, and protects against alkaliptosis. **D** In pyroptosis, lipopolysaccharide (LPS)-induced type I interferons (IFNA1 and IFNB1) activate IFNAR1/2 signaling, leading to histone deacetylation at the *CYP51A1* promoter and repression of its expression. Lanosterol accumulation alters membrane fluidity and suppresses STAT1/STAT2 activity, alleviating pyroptotic sensitivity.

Lipid metabolism plays a pivotal role in determining cell fate by integrating metabolic status with stress responses and cell death signaling pathways. In addition to serving as structural components of cellular membranes and energy stores, lipids and their metabolites function as bioactive signaling molecules that regulate multiple forms of regulated cell death. Enzymes involved in lipid biosynthesis—such as CYP51A1—are increasingly recognized as key modulators of cell survival and death, linking lipid homeostasis to the pathogenesis of diverse diseases (Fig. 3).

### Apoptosis

Apoptosis is a form of programmed cell death that plays a fundamental role in embryonic development, tissue homeostasis, and the elimination of damaged or transformed cells. It is also a principal mechanism by which many chemotherapeutic agents exert cytotoxic effects on cancer cells [52]. Apoptosis can be initiated via two major pathways: the intrinsic (mitochondria-dependent) pathway, which is triggered by cellular stress and mitochondrial outer membrane permeabilization, and the extrinsic (death receptor-mediated) pathway, both of which converge on the activation of caspases to execute cell death [53].

Accumulating evidence suggests that cholesterol and its metabolic intermediates influence cancer progression and immune regulation by remodeling the tumor microenvironment (TME). For example, in colorectal cancer (CRC), tumor-derived lanosterol can be taken up by CD8⁺ T cells, where it suppresses the mevalonate pathway and reduces prenylation and activity of KRAS proto-oncogene, GTPase (KRAS), ultimately promoting T cell apoptosis and impairing their antitumor function. Mechanistically, CRC cells with high levels of dual specificity phosphatase 18 (DUSP18) stabilize the transcription factor upstream stimulatory factor 1 (USF1), which upregulates SREBF2, thereby increasing lanosterol biosynthesis (a substrate of CYP51A1) and its release into the TME. Notably, pharmacological inhibition of DUSP18 with lumacaftor, in combination with anti-PD-1 (programmed cell death 1 (PDCD1; also known as PD-1) immunotherapy, significantly suppressed tumor growth in vivo [54]. Conversely, SKF 104976, a specific inhibitor of CYP51A1, induces apoptosis in human leukemia cells by depleting intracellular cholesterol. This apoptotic effect can be rescued by cholesterol supplementation but not by lanosterol, highlighting the functional importance of downstream cholesterol synthesis in cell survival [7].

These findings highlight a critical intersection between cholesterol metabolism, immune regulation, and cell death in cancer. Dysregulation of CYP51A1 activity may contribute to tumor progression by altering apoptotic sensitivity and shaping an immunosuppressive tumor microenvironment.

### Ferroptosis

Ferroptosis is a form of regulated necrosis characterized by iron-dependent lipid peroxidation and membrane rupture, driven by redox imbalance [55, 56]. Susceptibility to ferroptosis can be modulated by targeting various metabolic processes, including fatty acid metabolism, amino acid metabolism, iron homeostasis, glutathione biosynthesis, and lipid remodeling [57]. Among these, cholesterol metabolism plays a context-dependent role in ferroptosis, with cholesterol and its intermediates either promoting or suppressing ferroptotic cell death depending on cellular and microenvironmental conditions.

In the tumor microenvironment of melanoma, for instance, cholesterol promotes the expression of CD36 on tumor-infiltrating CD8⁺ T cells, leading to increased fatty acid uptake, lipid peroxidation, and ferroptosis. This impairs cytotoxic cytokine production and weakens antitumor immunity [58]. Similarly, in pancreatic ductal adenocarcinoma (PDAC) and NALM-6 leukemia cells, membrane lipid remodeling involving polyunsaturated fatty acid–cholesterol esters (PUFA-CEs)—generated via the acyl-CoA synthetase long-chain family member 4 (ACSL4)–sterol o-acyltransferase 1 (SOAT1) pathway and regulated by solute carrier family 47 member 1 (SLC47A1)—drives ferroptotic sensitivity [59]. Conversely, high intracellular cholesterol levels in long-term hematopoietic stem cells (LT-HSCs) support their survival and myeloid lineage bias under stress conditions such as irradiation. This protective effect is mediated through the solute carrier family 38 member 9 (SLC38A9)-mechanistic target of rapamycin kinase (MTOR) axis, which coordinates cholesterol sensing and signaling with the upregulation of solute carrier family 7 member 11 (SLC7A11) and glutathione peroxidase 4 (GPX4), key ferroptosis suppressors [60].

In addition to cholesterol, its intermediates also modulate ferroptosis. For example, 7-dehydrocholesterol (7-DHC), a product of sterol-C5-desaturase (SC5D), has antioxidant properties that suppress phospholipid peroxidation and ferroptosis in HEK-293T cells. In contrast, the conversion of 7-DHC to cholesterol by DHCR7 promotes ferroptosis, highlighting the therapeutic potential of targeting SC5D or DHCR7 in cancer and ischemia-reperfusion injury [50]. Several cholesterol biosynthesis enzymes—methylsterol monooxygenase 1 (MSMO1), CYP51A1, and EBP cholestenol delta-isomerase (EBP)—have been found to suppress ferroptosis similarly to SC5D, suggesting a broader protective role of the cholesterol biosynthetic pathway. However, lanosterol, the direct substrate of CYP51A1, does not affect RSL3-induced ferroptosis in HEK-293T cells [50]. In multiple myeloma (MM), interaction with bone marrow stromal cells (BMSCs) via CD40–CD40 ligand (CD40LG) signaling increases iron accumulation and activates the steroid biosynthesis pathway in MM cells, leading to enhanced lanosterol production. This, in turn, elevates reactive oxygen species (ROS) levels and sensitizes MM cells to ferroptosis induced by RSL3 treatment [61].

These findings suggest that cholesterol and its intermediates can play dual, context-specific roles in the regulation of ferroptosis. Importantly, targeting cholesterol biosynthetic enzymes such as CYP51A1 can influence ferroptosis independently of metabolite levels, implying that these enzymes may have non-canonical, cholesterol-independent functions in ferroptotic regulation.

### Alkaliptosis

Alkaliptosis is a distinct form of pH-dependent regulated cell death, mechanistically and morphologically different from apoptosis. It is triggered by the small-molecule compound JTC801, which selectively induces intracellular lethal alkalinization without altering extracellular pH, and is characterized by disruption of lysosomal pH homeostasis [62, 63]. Notably, alkaliptosis does not affect extracellular pH [63]. Alkaliptosis has demonstrated potent antitumor activity, particularly in PDAC, and offers a promising therapeutic strategy for malignancies resistant to conventional therapies [63–66].

Lysosomal integrity is critical for maintaining cellular homeostasis and viability, depending on both membrane stability and the function of membrane-associated transport proteins [67]. Cholesterol regulates membrane fluidity and stability and has been implicated in modulating susceptibility to alkaliptosis by altering lysosomal membrane dynamics. Mechanistically, JTC801 disrupts lysosomal pH, leading to cholesterol accumulation and activation of SREBF2, a master transcriptional regulator of sterol biosynthesis. This induces upregulation of CYP51A1, which in turn facilitates the metabolism of lanosterol—its substrate. Lanosterol has been found to inhibit transmembrane protein 175 (TMEM175), a lysosomal proton export channel, and its clearance via CYP51A1-dependent metabolism restores TMEM175 function, thereby dampening lysosomal alkalinization and mitigating the induction of alkaliptosis [68].

These findings raise several important open questions: Does CYP51A1 regulate alkaliptosis through mechanisms beyond its canonical role in lanosterol metabolism? Could its potential non-metabolic functions or subcellular localization—such as within lysosomes or other organelles—modulate lysosomal pH homeostasis and death susceptibility in a context- or tumor-type-specific manner? Elucidating these unanswered aspects will be critical for understanding how CYP51A1 contributes to alkaliptosis and for developing novel therapeutic interventions that target this pathway.

### Pyroptosis

Pyroptosis is a form of lytic regulated cell death mediated by the gasdermin protein family [69]. In the canonical pyroptotic pathway, pathogen- or danger-associated stimuli activate inflammasomes, which in turn activate caspase-1. Caspase-1 cleaves gasdermin D (GSDMD), whose N-terminal fragment forms pores in the plasma membrane, leading to cell lysis and the release of proinflammatory cytokines such as interleukin 1 beta (IL1B), IL-18, and IL1A [70]. Pyroptosis plays a role in containing infections and promoting immune clearance, but excessive or dysregulated pyroptosis has also been implicated in the pathogenesis of sterile inflammatory and autoimmune diseases. Macrophages are key executors of pyroptosis; they integrate diverse inflammatory signals and modulate gene expression programs to balance antimicrobial defense and tissue homeostasis [71].

Cholesterol and its metabolic intermediates are increasingly recognized as modulators of macrophage immune function, including their capacity for inflammatory signaling. For example, toll like receptor 4 (TLR4)-activated macrophages show increased expression of histone deacetylase 1 (HDAC1), a response dependent on type I interferon (IFN) signaling. Type I IFNs act as a secondary signal to repress Cyp51a1 expression, leading to the accumulation of lanosterol, a precursor in the cholesterol biosynthetic pathway. Pharmacological inhibition (e.g., with ketoconazole) or genetic knockdown of *Cyp51a1* in mouse macrophages similarly leads to lanosterol accumulation. This accumulation dampens IFNB1-mediated signaling, reducing activation of downstream transcription factors signal transducer and activator of transcription 1 (STAT1), STAT2, and IFN-stimulated genes. At the same time, lanosterol accumulation enhances membrane fluidity and increases ROS production, thereby boosting phagocytic activity, bacterial clearance, and survival in murine models of endotoxin shock [34].

These observations suggest that CYP51A1, through its regulation of sterol intermediates such as lanosterol, may modulate the inflammatory signaling landscape in macrophages and indirectly influence pyroptotic sensitivity. While direct evidence linking CYP51A1 to pyroptosis is currently lacking, its emerging role in shaping innate immune responses highlights the need to further investigate whether CYP51A1 functions as a metabolic checkpoint that fine-tunes inflammasome activation and pyroptotic outcomes in inflammatory diseases.

## CYP51A1 in diseases

**Fig. 4:**
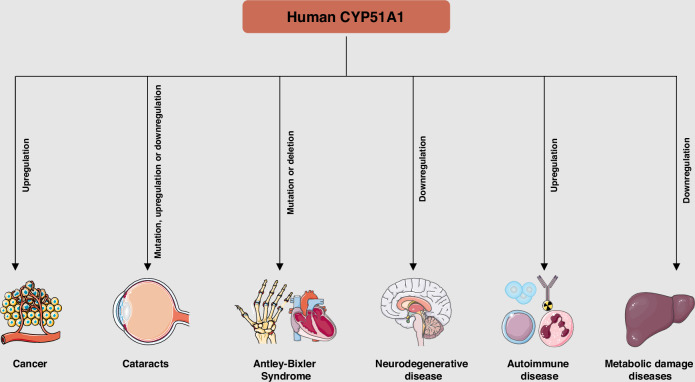
Dysregulation of CYP51A1 expression is associated with multiple human diseases. This schematic illustrates the diverse pathological outcomes associated with altered CYP51A1 expression across multiple human diseases. In cancer, elevated expression of CYP51A1 facilitates increased cholesterol biosynthesis, thereby supporting tumor growth and progression. In cataracts, either mutation or dysregulated expression of CYP51A1 may interfere with cholesterol homeostasis during lens development, potentially contributing to opacification. In Antley–Bixler syndrome, loss-of-function mutations or gene deletions impair sterol biosynthesis, resulting in congenital malformations such as skeletal and cardiac defects. In neurodegenerative diseases, such as Parkinson’s disease, decreased CYP51A1 expression reduces lanosterol levels, which may compromise neuronal survival. In autoimmune disorders, CYP51A1 upregulation has been linked to abnormal immune cell activation and impaired remyelination. In metabolic liver diseases, CYP51A1 downregulation contributes to cholesterol deficiency and promotes hepatic injury. Collectively, these disease associations underscore the critical role of CYP51A1 in maintaining tissue-specific metabolic balance and preventing pathogenesis.

Mutations or dysregulated expression of human CYP51A1 are implicated in diverse diseases, including cancer, genetic syndromes, neurological disorders, metabolic liver disease and immune dysfunction (Fig. 4). This section explores the underlying mechanisms by which aberrant CYP51A1 contributes to disease pathogenesis (Table 2).

**Table 2:** The role of aberrant CYP51A1 expression in diseases.

Diseases	Expression and role	Mechanism	Model	Refs.
Cancer	Upregulation; Promotion	Providing raw materials required for biofilm synthesis in cancer cells or remodeling the tumor microenvironment via the substrate lanosterol	Various cancer cell and mice	[68, 76, 77]
Cataracts	Mutation, upregulation or downregulation; Promotion	Mechanism unknown, may involve cholesterol metabolism disorder	Mice and patients	[47, 79, 80]
Antley-Bixler syndrome	Mutation or deletion; Promotion	Accumulation of endogenous substrates and lack of products	Mice	[35, 85]
Neurodegenerative disease	Upregulation; Inhibition	Increase lanosterol and delay disease progression by inducing mild mitochondrial depolarization and promoting autophagy	Cos-7 and A549 cells; mice	[87, 88]
Autoimmune disease	Upregulation; Promotion	Accumulation of endogenous substrates and lack of products	Oligodendrocyte; mice	[33, 90–92]
Metabolic damage diseases	Downregulation; Promotion	Cholesterol deficiency	Mice	[95]

### Cancer

Cancer is a multifactorial disease driven by interconnected biological processes [72]. Among these, metabolic reprogramming is not merely a byproduct of tumorigenesis but a selective adaptation that supports tumor growth and progression [73–75]. For example, microsatellite stable colorectal cancer (MSS CRC) cells accumulate distal cholesterol precursors (DCPs) due to asynchronous cholesterol biosynthesis, polarizing T cells toward a pro-tumorigenic Th17 phenotype and promoting immune suppression. Inhibition of CYP51A1, either pharmacologically (e.g., with ketoconazole) or genetically, reduces DCP accumulation, reprograms the immune microenvironment, and suppresses MSS CRC progression. Clinical data from large cancer cohorts further support the presence of dysregulated cholesterol biosynthesis in MSS CRC [5]. Similarly, lanosterol, a key intermediate in cholesterol synthesis, can inhibit CD8⁺ T cell activation by disrupting KRAS–mitogen-activated protein kinase (MAPK) signaling, though the specific role of CYP51A1 in this context remains to be defined [54].

Sterol biosynthesis also influences therapy response. Inactivation of MSMO1 or its partner NAD(P) dependent steroid dehydrogenase-like (NSDHL) sensitizes various tumor cell lines (such as A431, SCC61, SCC68, SCC25, PC9, FaDu, Detroit and MCF12F) to epidermal growth factor receptor (EGFR) inhibitors by promoting EGFR degradation via lysosomal trafficking. Notably, metabolic inhibition with ketoconazole or *CYP51A1* knockdown can reverse this sensitivity, suggesting a context-dependent role for CYP51A1 in treatment resistance [76]. Additionally, the transcription factor YY2 has been identified as an endogenous repressor of CYP51A1 and can suppress cholesterol biosynthesis and tumor progression in hepatocellular carcinoma [77].

Thus, CYP51A1-regulated cholesterol intermediates can function as immunometabolic signals that shape both tumor cell behavior and the immune landscape. Deciphering how CYP51A1-mediated sterol metabolism impacts the tumor and its microenvironment may reveal new therapeutic strategies for cancer prevention and treatment.

### Cataracts

Hereditary pediatric cataract is a rare Mendelian disorder characterized by lens opacity, leading to visual impairment or blindness [78]. Numerous gene mutations have been implicated in its pathogenesis, including mutations in *CYP51A1*.

In a cohort study of 38 children with congenital cataracts, a missense mutation in *CYP51A1* was identified in one family [79]. Retrospective analysis revealed that affected individuals exhibited nonspecific congenital cataracts but had otherwise normal neurological and systemic function [80]. Another study investigating 115 cataract-associated genes in 36 unrelated patients with syndromic or nonsyndromic bilateral congenital cataracts found one patient harboring compound heterozygous mutations in *CYP51A1* (I312T and Y421X, rs141654764). This patient exhibited nuclear and lamellar opacities, developmental delay, spastic hemiparesis, and cryptogenic neonatal cirrhosis, suggesting a broader pathogenic role for *CYP51A1* mutations beyond isolated cataract formation [81].

In support of this, patients carrying the L232P mutation in *CYP51A1* presented with congenital cataracts, neonatal cholestatic jaundice, elevated liver enzymes, and hyperferritinemia, indicating that *CYP51A1* mutations may underlie a syndromic disorder affecting both ocular and hepatic systems. Moreover, dysregulation of Cyp51a1 expression during lens development has been observed in mouse models of multisystem disorders. Specifically, Cyp51a1 expression is decreased in SWI/SNF related BAF chromatin remodeling complex subunit ATPase 4 (SMARCA4) and E2F transcription factor 1/2/3 (*E2f1/2/3*) knockout mice, while increased in tudor domain containing 7 (*Tdrd7*), KLF transcription factor 4 (*Klf4*), MAF BZIP transcription factor G (*Mafg*), and *Mafk* knockout models, further supporting its role in lens development and cataractogenesis [47].

The presence of *CYP51A1* mutations in patients with congenital cataracts—often accompanied by hepatic or neurological symptoms—suggests its dual role in ocular development and systemic homeostasis. Given the genetic heterogeneity of pediatric cataracts, further integrative genomic studies are warranted to define the precise contribution of CYP51A1 to lens development and to explore its potential as a diagnostic marker or therapeutic target in congenital cataract syndromes.

### Antley-Bixler Syndrome

Human Antley-Bixler Syndrome (ABS) is a heterogeneous group of disorders characterized by skeletal, cardiac, and genitourinary abnormalities [82]. CYP51A1-mediated cholesterol metabolism affects the synthesis of sex hormones and glucocorticoids, which can lead to heart defects and skeletal abnormalities [83, 84].

For example, specific deletion of cytochrome P450 oxidoreductase (*POR*) in mouse limb bud mesenchymal cells results in short forelimbs and hindlimbs, thin skeletal elements, and fused joints. Transcriptional analysis of forelimb buds from *E12.5* mice showed that expression of genes in the entire cholesterol biosynthesis pathway was upregulated in the limbs of conditional knockout (CKO) mice, and that cholesterol deficiency explained most of the phenotype. Thus, POR-dependent cholesterol synthesis is essential during limb and bone development. Whereas the presence of point mutations in *POR* in ABS patients can lead to limb-skeletal defects [85]. Indeed, ABS is associated with impaired aberrant steroidogenesis, which is largely attributed to impaired POR-dependent CYP51A1, CYP17A1, CYP21A2, and CYP19A1 activities [86]. Similarly, *Cyp51a1*^*-/-*^ knockout mice die around embryonic day 15, and the embryos exhibit several developmental malformations similar to ABS, such as skeletal malformations and heart defects. *Cyp51a1*^*-/-*^ embryos exhibit upregulation of all cholesterol-producing genes except *Dhcr7*, and cholesterol synthesis is completely blocked, while lanosterol and 24,25-dihydrolanosterol accumulate [35].

These findings highlight CYP51A1 as a critical regulator of embryonic development, particularly in tissues reliant on tightly controlled cholesterol biosynthesis such as the skeletal and cardiovascular systems. The embryonic lethality and ABS-like malformations observed in Cyp51a1⁻/⁻ mouse models underscore the enzyme’s non-redundant role in developmental steroidogenesis and sterol homeostasis. These models provide a valuable platform to further dissect the pathomechanisms of ABS and may inform therapeutic strategies targeting sterol biosynthesis defects in congenital disorders.

### Neurodegenerative disease

Unlike its role in cancer, where lanosterol accumulation can suppress immune responses and promote tumor progression, lanosterol may exert protective effects in non-malignant conditions, particularly in neurodegenerative diseases. These disorders, characterized by the progressive loss of neuronal structure and function, include movement disorders such as cerebellar ataxia and cognitive disorders such as Alzheimer’s disease, Parkinson’s disease, and Huntington’s disease. Emerging evidence suggests that modulating CYP51A1 activity in the brain to increase lanosterol levels may represent a novel neuroprotective strategy.

For example, in a Parkinson’s disease mouse model treated with 1-methyl-4-phenyl-1,2,3,6-tetrahydropyridine (MPTP), lanosterol levels were reduced by approximately 50% in the nigrostriatal region, a key site of dopaminergic neuron loss. Supplementation with exogenous lanosterol rescued dopaminergic neurons by inducing mild mitochondrial depolarization and activating autophagy, thereby reducing neuronal cell death [87]. Similarly, lanosterol treatment decreased the aggregation of neurotoxic proteins in Cos-7 and A549 cells through autophagy enhancement [88]. In support of a broader role in neurodevelopmental disorders, whole-genome sequencing of 327 children with cerebral palsy and their parents identified *CYP51A1* as a plausible candidate gene linked to the disease [89].

These findings suggest that CYP51A1-mediated sterol metabolism, particularly the regulation of lanosterol levels, may play a beneficial role in protecting against neuronal degeneration. Targeting CYP51A1 could therefore offer a promising sterol-based therapeutic approach for neurodegenerative and neurodevelopmental disorders.

### Autoimmune disease

Autoimmune diseases arise from the breakdown of immune tolerance, whereby the immune system mounts aberrant responses against self-antigens, leading to tissue damage. Recent evidence suggests that CYP51A1, a key enzyme in cholesterol biosynthesis, may influence immune cell function and thereby contribute to autoimmune disease development. In a mouse model of Sjögren’s syndrome, pharmacological inhibition of Cyp51a1 with ketoconazole suppressed CD4⁺ T cell activation and proliferation, reduced T cell infiltration in salivary gland lesions, and alleviated disease symptoms [33]. These findings suggest that CYP51A1 may regulate immune activation by modulating sterol intermediates that influence T cell function.

In multiple sclerosis (MS), a neuroinflammatory disease driven by immune-mediated destruction of myelin-producing oligodendrocytes, CYP51A1 has emerged as a potential therapeutic target. Hubler et al. identified CYP51A1, along with transmembrane 7 superfamily member 2 (TM7SF2) and EBP, as regulators of oligodendrocyte differentiation through a high-throughput chemical screen. Inhibiting these enzymes promoted remyelination in vivo by depleting toxic 8,9-unsaturated sterol intermediates, which otherwise block oligodendrocyte maturation [90, 91]. Furthermore, transcriptomic profiling of CD4⁺ T cells from cerebrospinal fluid versus blood in MS patients identified CYP51A1 as a candidate gene implicated in disease-specific immune responses [92].

These findings highlight a novel link between CYP51A1-mediated sterol metabolism and the regulation of both immune and neural cell function. By influencing CD4⁺ T cell activation and oligodendrocyte maturation, CYP51A1 may serve as a metabolic checkpoint in autoimmune pathogenesis. However, the etiology of autoimmune diseases is multifactorial, involving genetic predisposition, environmental triggers, and complex immune networks. Thus, while targeting CYP51A1 may offer therapeutic benefit, it is unlikely to be sufficient alone to fully restore immune tolerance and homeostasis.

### Metabolic liver disease

The liver is a central metabolic organ responsible for the regulation of carbohydrate, lipid, and protein metabolism [93]. Disruption of key metabolic enzymes in the liver, such as CYP51A1, can profoundly alter hepatic physiology and systemic homeostasis. In mice, hepatocyte-specific knockdown of *Cyp51a1* impairs cholesterol biosynthesis, leading to hepatomegaly, oval cell hyperplasia, fibrosis, and inflammation. These pathological features are associated with a reduction in cholesteryl esters, resulting in cell cycle arrest and the induction of an aging-associated secretory phenotype (SASP). Interestingly, sex-specific differences emerged: females exhibited more pronounced downregulation of metabolic pathways, while males showed stronger immune activation. Dietary interventions revealed that dietary fat mitigated liver injury predominantly in females, whereas dietary cholesterol reversed hepatic fibrosis in both sexes—highlighting cholesterol deficiency as a key driver of sex-dependent liver injury [94].

Similarly, developmental *Cyp51a1* knockout in mice leads to progressive hepatic injury beginning at puberty, with ductular reactions and fibrosis more severe in females. These differences may be attributed to reduced signaling through the retinoic acid receptor-related orphan receptor C (RORC), whose activity depends on endogenous sterol ligands produced by CYP51A1 [95]. Paradoxically, pharmacological inhibition of CYP51A1 using CP-320626 in dogs and obese rodent models lowers plasma cholesterol and improves hyperglycemia and dyslipidemia, suggesting potential therapeutic benefit in type 2 diabetes [96]. Thus, targeting CYP51A1 has content-dependent effects depending on the biological context and the presence or absence of underlying disease.

Overall, the pathological outcomes associated with CYP51A1 dysregulation are complex and multifactorial, involving impaired enzyme function, metabolic disruption, immune and hormonal imbalances, and altered responses to pharmacologic interventions. Continued investigation into the structure, regulation, and context-specific functions of CYP51A1 will be essential for understanding its role in liver pathology and for developing targeted therapies for metabolic, hepatic, and systemic diseases.

## Targeting CYP51A1: small-Molecule inhibitors and therapeutic potential

The cholesterol biosynthetic pathway has proven to be a successful target in drug development, as exemplified by statins for hypercholesterolemia and azole antifungals for infectious diseases [97, 98]. Several small-molecule inhibitors of CYP51A1 have been developed, including clinically used azoles such as fluconazole and ketoconazole, which were originally designed to target fungal CYP51A1 but also inhibit the human enzyme (Table 3).

**Table 3:** Selected small-molecule inhibitors targeting CYP51A1.

Name	Status	Mechanism	Refs.
Ketoconazole	Approved, Investigational	Inhibitor of cyclosporine oxidase and testosterone 6 beta-hydroxylase, cytochrome P450 C17 inhibitor	[124]
Miconazole	Approved, Investigational, Vet_approved	Antifungal agent, imidazole antifungal agent	[125]
Posaconazole (SCH 56592)	Approved, Investigational, Vet_approved	Sterol C14ɑ demethylase inhibitor	[126]
Voriconazole	Approve	CYP51 inhibitor	[127]
Fluconazole	Approved, Investigational	Triazole antifungal agent	[128]
Tioconazole	Approved	antifungal medication	[129]
Itraconazole	Approved, Investigational	antifungal agent	[130]
VT-1161	Approved, Investigational	CYP51 inhibitor, antifungal	[131]
Ergosterol	Approved, Experimental	Inhibition of CYP51 enzyme activity to exert its antifungal effect	[132]
VFV, Azalanostat, LK-935, SKF 104976	/	CYP51 inhibitor	[7, 21, 100, 101]

Ketoconazole, in particular, has been widely used to inhibit CYP51A1 in mammalian systems. Inhibition of CYP51A1 with ketoconazole has demonstrated diverse biological effects: it rescued EGFR degradation in A431 carcinoma cells by blocking sterol metabolism upstream of MSMO1, and reduced inflammatory signaling and cytokine release in mouse macrophages, ultimately improving survival in a model of endotoxic shock. However, its broad inhibitory activity may cause drug-drug interactions and hepatotoxicity, as ketoconazole can impair the metabolism of other co-administered compounds. Notably, ketoconazole abolished low-density lipoprotein (LDL)-induced reductase activity at submicromolar concentrations in various mammalian cell lines (IEC-6 cells, skin fibroblasts, HepG2, and Chinese hamster ovary cells), suggesting a potential application in cardiovascular disease [99].

Despite the use of fungal CYP51A1 inhibitors in preclinical models, their applicability to human diseases is limited due to structural differences between human and microbial CYP51A1. In a study screening commercial and experimental CYP51A1 inhibitors for anti-cancer activity, only VFV, a compound with a bulky imidazole-oxadiazole core, showed potent anti-proliferative effects across multiple cancer cell types. Crystal structures of the human CYP51A1–VFV complex revealed a 2:1 inhibitor-to-enzyme binding ratio, explaining the enzyme’s broader substrate profile and reduced sensitivity to inhibition compared to fungal homologs [100].

Other synthetic compounds have also shown promise. Azalanostat (RS-21607), an imidazole derivative, inhibited cholesterol synthesis in HepG2, fibroblasts, and rodent hepatocytes and holds potential in atherosclerosis prevention [101]. Likewise, LK-935, a pyridylethanol(phenylethyl)amine derivative, binds to the heme pocket of CYP51A1, similar to azoles, and suppresses sterol synthesis in hepatoma cells [21]. SKF 104976 treatment of HL-60 leukemia cells in cholesterol-depleted media inhibited cell division and induced polyploidy, underscoring its antiproliferative potential.

Endogenous sterol metabolites also modulate CYP51A1 activity. 25-hydroxycholesterol, a negative regulator of cholesterol biosynthesis, downregulates *CYP51A1* mRNA in adrenocortical H295R and HepG2 cells. Intriguingly, cholesterol deprivation upregulates CYP51A1 in H295R but not in HepG2 cells, suggesting cell-type-specific regulatory mechanisms that could be leveraged to suppress hepatocellular carcinoma growth [17].

While CYP51A1 overexpression is frequently associated with cancer progression, emerging strategies explore the potential therapeutic benefit of its hyperactivation under select conditions. For example, inhibition of protein phosphatase 2 activator (PTPA) in colorectal cancer leads to activation of oncogenic pathways (e.g., MAPK, catenin beta 1 [CTNNB1]) and cellular stress responses. Subsequent inhibition of WEE1 G2 checkpoint kinase (WEE1) kinase results in synthetic lethality, highlighting how manipulating upstream or downstream pathways of CYP51A1 may promote selective cancer cell death [102]. These findings open the possibility that activators of CYP51A1, rather than inhibitors, could be exploited under specific oncogenic contexts.

While several CYP51A1 inhibitors have shown promise in preclinical and clinical settings, their use in humans remains limited by off-target effects, structural specificity, and adverse interactions. The development of highly selective and structurally optimized CYP51A1 inhibitors—or context-specific modulators—could provide valuable therapeutic strategies, particularly in hormone-sensitive cancers, immune regulation, and metabolic diseases. Future efforts should focus on improving target specificity, minimizing toxicity, and exploring the dual potential of both inhibition and activation of CYP51A1 depending on disease context.

## Conclusion and future perspectives

As a core enzyme in the cholesterol biosynthesis pathway, CYP51A1 has long been recognized for its role in catalyzing lanosterol demethylation—an evolutionarily conserved step across species. However, growing evidence now positions CYP51A1 as far more than a metabolic workhorse. Emerging data reveal that CYP51A1 influences diverse biological processes, including cell proliferation, immune regulation, and multiple forms of regulated cell death such as apoptosis, ferroptosis, alkaliptosis, and potentially pyroptosis. These roles are mediated not only through cholesterol end-products but also through intermediate sterol metabolites that act as signaling molecules within distinct tissue and disease contexts.

Dysregulation of CYP51A1 has been implicated in a spectrum of human diseases—from cancer, congenital cataracts, and autoimmune conditions to neurodegenerative and liver diseases. Its function appears to be both context- and cell-type-dependent, sometimes promoting survival and at other times enhancing susceptibility to death or dysfunction. Intriguingly, CYP51A1 also contributes to tumor immune evasion, remyelination in the CNS, and sex-specific liver injury, underscoring its diverse and systemic influence on human physiology and pathology.

Despite these advances, several critical questions remain unresolved. How does CYP51A1 modulate cell death pathways independently of cholesterol synthesis? What are the specific sterol intermediates or non-metabolic functions that confer its regulatory versatility? How do tissue-specific regulators, subcellular localization, or post-translational modifications shape its biological activity? Addressing these questions will be essential for fully understanding CYP51A1’s role as a metabolic checkpoint in both normal and disease states.

Therapeutically, targeting CYP51A1 presents both challenges and opportunities. While broad-spectrum inhibitors such as azole antifungals have demonstrated efficacy, they are limited by off-target effects and drug–drug interactions. The future lies in developing selective modulators—both inhibitors and potential activators—tailored to specific diseases and biological contexts, such as hormone-sensitive cancers, neuroinflammatory conditions, and metabolic syndromes.

In conclusion, CYP51A1 stands at the intersection of metabolism, cell fate, and disease, representing a promising yet underexplored target for therapeutic innovation. Ongoing research into its molecular regulation, functional diversity, and clinical relevance will not only advance our understanding of sterol biology but also unlock new avenues for precision medicine.
